# Determining the effect of selected mental factors on turnover intention through two modulators - stress and resilience over COVID-19 period

**DOI:** 10.1186/s12913-023-09268-z

**Published:** 2023-04-14

**Authors:** Seyed mahdi mousavi, Saeid Yazdanirad, Mahsa Jahadi naeini, Amirhossien khoshakhlagh, Mojtaba Haghighat

**Affiliations:** 1 Student Research Committee, Department of Occupational Health and Safety Engineering, School of Health, Isfahan University of Medical Sciences, Isfahan, Iran; 2 Social Determinants of Health Research Center, Shahrekord University of Medical Sciences, Shahrekord, Iran; 3 School of Health, Shahrekord University of Medical Sciences, Shahrekord, Iran; 4 Department of Occupational Health Engineering, School of Public Health, Isfahan University of Medical Sciences, Isfahan, Iran; 5 Department of Occupational Health Engineering, Faculty of Health, Kashan University of Medical Sciences, Kashan, Iran; 6 Behbahan Faculty of Medical Sciences, Behbahan, Iran

**Keywords:** Mental factors, Turnover intention, Resilience, Job stress, General health, Mental workload, Work-family conflict, Fear of COVID-19

## Abstract

**Introduction:**

Turnover intention among nurses has risen in an alarming rate since the onset of the pandemic. There are various underlying factors to turnover intention. The present study aims to determine the effect of a number of mental factors on nurses’ professional-turnover intention through two modulators of stress and resilience over COVID-19 period.

**Methods:**

The current cross-sectional study was conducted at three hospitals in Khuzestan Province, southern Iran, during the winter of 2021. To collect the data, given the restrictions in place during COVID-19 period, the web link of electronic self-reported questionnaires (including general health, mental workload, work-family conflict, resilience, job stress, corona fear, and turnover intention) were sent to 350 nurses through e-mail and other social media (WhatsApp and Telegram). Accordingly, they were asked to complete the questionnaire during rest periods within two weeks. Totally, 300 people (85% participation) filled out the questionnaires. Finally, a model was constructed in the Amos software.

**Results:**

The results showed that the four independent parameters of decreasing general health, increasing mental workload, increasing WFCs and fear of COVID-19 can indirectly increase nurses’ turnover intention by increasing job stress. Among these variables, the highest indirect effect coefficient on turnover intention was related to the general health parameter (-0.141). The results also demonstrated a negative correlation between job stress and resilience, with lower resilience raising job stress and, consequently, increasing intention to quit the job.

**Conclusion:**

Mental factors affecting turnover intension were identified in this study through path analysis. Therefore, it is recommended that the required resilience-enhancing measures to be taken by hospitals and nursing administrations to reduce psychological pressures caused by mentioned variables with the aim of minimizing job-related stress and fostering nurse retention.

## Introduction

It was on March 11, 2020 that the World Health Organization announced that a new strain of the coronavirus family named SARS-COV-2 was associated with an outbreak termed COVID-19 [1]. The disease spread rapidly through the world, killing a large number of people due to its high contagiousness and lack of definitive treatment [2]. Hence, since the containment of this pandemic and treatment of patients have been a top priority, many countries’ healthcare systems and in turn healthcare providers were left overstretched and overwhelmed. In this regard, the pivotal role of healthcare workforce has been emphasized more than ever. The health care workers have crucial task to save human lives, particularly in the conditions of the COVID-19 pandemic and, hence, special attention to their health and wellbeing can directly reflect on the patients’ safety and health [3].

Fighting in the frontline of battle against the COVID-19 pandemic, nursing carried a heavy burden during this pandemic [4]. The factors such as critical shortage of specialized personnel, fatigue, emotional trauma, ethical dilemma, fear of contracting COVID-19 and subsequent transmission of virus to loved ones, and affected work-life balance are among those faced by nurses [5, 6]. Therefore, despite experiencing physiological complications, the mentioned factors can cause elevated psychological disorders such as stress, fear, anxiety in nurses [7]. Consequently, nurses have been more likely to develop adverse mental health consequences than other healthcare professionals [8, 9].

Evidence of systematic review from the previous outbreaks used to explore the potential impact of COVID-19 on mental health outcomes of health-care providers and the implications for service solutions, revealed that levels of moderate anxiety ranged from 22.6 to 44.6%, and severe anxiety from 2.9 to 5.3% [10]. Moreover, 34% of health-care workers experienced mild depression, 22% moderate depression, and 6.2% had severe depression [11].

Several epidemiological studies have corroborated the prolonged effects of COVID-19 on the psychological health of nurses. COVID-19 can decrease the retention rate and increase the turnover retention among nurses. Furthermore, turnover intention among nurses has risen in an alarming rate since the onset of the pandemic [12]. Specifically, employee turnover is considered as the number of employees who leave an organization over a specified timeframe, typically one year. On the other hand, employee retention is the number of staff an organization manages to keep employed during a given period.

According to various researches, prior to the outbreak of COVID-19, 15 to 44% of nurses reported their intention to quit their jobs worldwide [13]. In Iran, similarly, the rate of turnover was reported to be 32.7 to 35% [13, 14]. However, this figure has not been officially specified during the COVID-19. Previous studies have also shown that among the most important factors influencing nurses’ high turnover are job stress, general health, resilience, and work-family conflicts [15, 16]. On the other hand, the fear of COVID-19 (FCV-19) can exacerbate the mentioned factors leading to a higher turnover rate [17].

Job stress is one of the factors that is directly related to the tendency to leave the service owing to an imbalance between the task and work load [18]. In this sense, Said & El-Shafei assessed job satisfaction, job stress and turnover intention among nurses during the Corona pandemic period in Egypt and found that 75% of nurses have varying levels of job stress and only 4% of them were inclined to continue their job [19].

The second factor associated with turnover intention in nurses is general health. The results of a study conducted before the outbreak of COVID-19 in Iran demonstrated that about 33% of nurses did not have a desired general health level. The general health of nurses is affected by factors such as low organizational support, shiftwork, high work load, prolonged working hours [20].

The next factor that affects nurses’ leaving intentions is work–family conflicts (WFCs). WFCs is a negative interaction between work and family duties in which the pressures of both are incompatible, and various expectations and roles do not match. In this case, if one cannot maintain a balanced work-family relationship, it leads to a conflict, which, in turn, places a significant burden on individual, family and organization [21, 22].

The amount of thinking, level of cognitive demand, or thought processing effort required by the worker to meet the physical, temporal, and environmental demands of the stated task is referred to as mental workload. Mental workload is a multidimensional concept with several facets [23]. The individual’s processing capacity and the task’s demands determine mental workload perception [24]. Various studies have shown that in jobs with excessive workload, due to fatigue and poor employee scheduling, efficiency decreases and reduced memory, damage to the thought process, irritability, and reduced learning ability are ensued [25]. Furthermore, over COVID-19 period, nurses’ heavy mental workload has reportedly been linked to elevated occupational stress, posing a risk to patients’ safety [26].

Another factor that can predict the nurses’ turnover intention is resilience in stressful and critical situations [27]. The results of previous studies show that increasing the resilience of individuals can be a way to deal with stressful situations [28]. Resilience is described as a person’s belief in their capacity to cope with stress and maintain emotional stability, and it is a factor that can help to mitigate the negative effects of a variety of physical and mental issues and illnesses [29, 30]. In this regard, Foster et al. acknowledged that, in order to cope with work challenges and maintain their mental health and stability, 21st-century nurses must acquire resilience skills [31] as a way of increasing mental health and job and life satisfaction [32].

Accordingly, the present study aims to determine the effect of mental factors on the turnover intention of nurses through two modulators of stress and resilience over COVID-19 period.

## Method

### Participants

The current cross-sectional study was conducted at three hospitals in Khuzestan province, southern Iran, during the winter of 2021.The inclusion criteria are as follows: having more than one year of work experience, not having chronic diseases such as cancer, AIDS, cardiovascular and MS, not having mental disorders, not taking psychiatric drugs. Participants who were not responded to the mail and participants who not filled the questionnaire or incompletely filled were excluded from the study. The sample size was determined using Cochran’s formula of 300 people. For selection of the samples, a list of the nurses employed in COVID-19 ward in these hospitals, including 823 nurses, was prepared. Then, 600 individuals were randomly selected from them. For this purpose, a number code was considered for each of them and the software was used to opt the individuals. After that, their medical records were reviewed. Following that, 523 nurses with inclusion criteria were entered the study. Then, these persons were called and invited to participate in the study. Among them, 350 nurses accepted to participate in the study. Finally, out of 350 responders in the study, 300 nurses filled out the questionnaires accordingly.

### Data collection

The protocol for performing this study was reviewed and approved by the ethics committee of Behbahan Faculty of Medical Sciences (code: IR.BHN.REC.1401.024). To collect data, given the conditions of the COVID-19 disease, the web link of questionnaires along with an instruction were sent to the subjects who accepted to participate in this study. The maximum time of two weeks were considered for completing the questionnaires. Ultimately, 300 completed questionnaires were obtained and statistically analyzed.

### Tools

To gather information for this investigation, seven questionnaires were used including general health questionnaire, mental workload questionnaire, work-family conflict questionnaire, resilience questionnaire, job stress questionnaire, corona fear questionnaire and turnover intention questionnaire, which are referred to as below.

#### Fear of COVID-19 scale (FCV-19 S)

The Corona Fear Questionnaire was developed by Ahorsu et al. in 2020 to measure people’s fear of COVID-19. This questionnaire includes seven items and is based on a five-point Likert scale. The scale is 1 to 5, with 1 being the lowest and 5 being the highest. As a result, each person’s score in this questionnaire ranges from 7 to 35, with higher values indicating greater dread of the corona virus. The correlation between these items has been reported from 0.66 to 0.74. The obtained Cronbach’s alpha coefficient is 0.82. Reliability values of the current Persian version of FCV-19 S questionnaire proved acceptable, with internal consistency (α = 0.82) and retest reliability (ICC = 0.72). Concurrent validity with hospital anxiety and depression scale (with depression, r = 0.425 and anxiety, r = 0.511) and perceived [33, 34].

#### Job stress questionnaire

This questionnaire consists of 60 questions that are equally divided in 6 dimensions of role workload, incompetent of role, role ambiguity, role boundary, responsibility and physical environment. The score of the OSIPOW Job Stress Questionnaire is based on a 5-point Likert scale. For each phrase, 5 options are never equal to 1 point, sometimes 2 points, often 3 points, usually 4 points and most of the time 5 points. The range of this questionnaire is between 60 and 300, with higher scores indicating high stress level. Total stress is divided into four categories: low stress (50–99 points), low to moderate stress (100–149), moderate to severe stress (150–199) and severe stress (200–250) [35]. Moreover, in the course of the research carried out by Sharifian et al., The validity and reliability of the Persian version of this questionnaire was assessed and its Cronbach’s alpha coefficient was calculated and reported 0.83 [36].

#### Turnover intention questionnaire

This turnover intention questionnaire developed by Kim et al. (2007) comprises of 15 questions and scores on a five-point Likert scale including Strongly Disagree (1), Disagree (2), No Comment (3), Agree (4) Strongly Agree (5). The lower limit of the individual score in this questionnaire is 15 and the upper limit is 75. Haji et al. validated the Persian version of this questionnaire in Iran, and its reliability was reported as 0.84, and its validity was confirmed [37].

#### General health questionnaire (GHQ)

The 28-item GHQ was first developed by Goldberg (1972) consists of 4 sub-tests, each of which has 7 questions. The questions of each sub-test are sequentially arranged, with questions 1 to 7 relating to somatic symptoms, 8 to 14 about anxiety and insomnia, 15 to 21 on social dysfunction, and 22 to 28 relating to the severe depression. All items of the general health questionnaire include 4 options. The Likert scoring method is based on test options scored as (1,2,3,4) and, as a result, the total score of an individual will vary from zero to 84. The scores of each subject in each scale are calculated separately and then added to the scores of 4 subscales to get the total score. A lower score indicates better mental health [38].

#### NASA-TLX mental workload questionnaire

The NASA-TLX method developed in 1988 by Hart and Staveland (1988) is a well-known tool for evaluating workload from an individual perspective. This is a multidimensional method that calculates a workload score based on a weighted average of six scales: temporal and mental demand, physical demand, performance, effort, and Frustration [39]. Validity and reliability of the Persian version of the Mental Worker Questionnaire was conducted by Mohammadi and his colleagues in 2013 [40].

#### Work-family conflict questionnaire

To measure work-family conflict, an 18-item multidimensional questionnaire on work-family conflict was used by Carlson et al. (2000). According to literature, there are three forms of work–family conflict including time-based conflict, strain-based conflict, and behavior-based conflict. In 1991, Gutek et al. defined two directions for each of these three forms of work–family conflict: (a) conflict due to work interfering with family (WIF) and (b) conflict due to family interfering with work (FIW). Hence, when combining three forms and two directions, it yields six dimensions of work–family conflict as follows: (1) time-based WIF, (2) time-based FIW, (3) strain-based WIF, (4) strain-based FIW, (5) behavior-based WIF, and (6) behavior-based FIW. The answers range from option 1 (strongly agree) to 5 (strongly disagree) using the Likert scale [41].

#### CD-RSC resilience questionnaire

Scoring in this 25-item questionnaire, designed by Davidson & Connor in 2003, is based on the Likert scale (completely incorrect score 0, rarely 1, sometimes true 2, often true 3, always the true score is 4. Therefore, the minimum score is 0 and the maximum is 100. The cut-off point of this questionnaire is 50 points. In other words, a score higher than 50 indicates good resilience, and the higher the score, the more the severity of resilience of the individual will be higher and on the contrary, the reliability of the questionnaire was reported by Connor and Davison 0.89 [42]. The reliability of the Persian version of the resilience questionnaire of Samani et al. Was done in 2007 [43].

#### Data analysis

The data were initially entered into the IBM SPSS version 26 software for analysis. The results indicated that the data distribution is normal after the normality of the variables was tested using skewness and elongation curves. In order to investigate the relationship between the variables under study, the Pearson test was applied. The correlations between the variables were then looked at after a model was constructed in the Amos software. The variables used in the data analyses included general health, mental workload, work – family conflict, fear of COVID-19, job stress, resilience, and turnover intentions. The model’s fit was assessed using fit indicators.

## Result

Of 350 nurses, 300 persons participated in this study and a participation rate of almost 86% was recorded. The mean and standard variation values in the participants were 42.15 and 9.46, respectively. The other demographic details of the study’s participants are shown in Table 1.

**Table 1:** Frequency distribution of demographic and occupational characteristics of participants

Variables		Frequency	Percentage
Age (year)	20 to 30	39	13.0
	30 to 40	63	21.0
	40 to 50	116	38.7
	More than 50	82	27.3
Sex	Male	175	58.3
	Female	125	41.7
Education level	Diploma	38	12.7
	Associate degree	34	11.3
	Bachelor of Science	194	64.7
	Master of Science	34	11.3
Shift work	Yes	179	59.7
	No	121	40.3
Marital status	Single	72	24.0
	Married	228	76.0
Job experience (year)	1–5	135	44.9
	6–10	80	26.6
	11–15	60	20
	More than 15	25	8.33
Second job	Yes	121	40.3
	No	179	59.6

The results showed that the average job stress in this study was equal to 154.29 ± 40.99. The total score of job stress is divided into four categories: low stress (50 to 99), low to moderate stress (100 to 149), moderate to severe stress (150 to 199) and severe stress (200 to 250). The mean value of job stress in this study was in the category of moderate to severe stress. Additionally, the Table 2 represents the maximum, minimum, mean, and standard variation values of the scores related to the studied variables. The mean value of the fear of COVID-19 among the nurses was equal to 26.09 (min = 7.00, max = 38.00, and SD = 7.99).

**Table 2:** frequency distribution of studied variables

Variables		Minimum	maximum	Mean	Standard deviation
Job stress	Role workload	1	50	16.68	9.50
	Incompetent of role	7	50	15.90	9.02
	Role ambiguity	8	50	16.04	10.18
	Role boundary	10	60	16.10	9.90
	Responsibility	10	70	42.39	15.82
	Total	60	260	154.29	40.99
Resilience	Total	25.00	100.00	70.70	23.02
Mental workload	Mental Demand	40	95	68.07	8.59
	Physical Demand	20	90	64.08	14.23
	Temporal Demand	30.00	95.00	64.08	15.21
	Performance	15.00	90.00	65.02	14.73
	Effort	25.00	95.00	67.86	11.80
	Frustration	35.00	95.00	72.33	12.61
	Total	46.67	82.50	66.90	5.96
General health	Somatic symptoms	0.00	21.00	20.40	3.04
	anxiety and insomnia	0.00	26.00	10.70	7.24
	Social dysfunction	0.00	26.00	8.15	6.70
	Severe depression	0.00	21.00	3.21	4.66
	Total	8.00	78.00	42.47	12.35
Turnover intentions	Total	15.00	75.00	59.39	13.72
Work – family conflict	Time-based work interference with family	3.00	15.00	10.01	3.63
	Time-based family interference with work	0.00	15.00	9.66	3.46
	Strain-based work interference with family	5.00	15.00	11.63	2.26
	Strain-based family interference with work	3.00	21.00	9.97	3.00
	Behavioral-based work interference with family	3.00	15.00	11.49	2.87
	Behavioral-based family interference with work	3.00	15.00	9.15	3.57
	Total score of work-family conflict	31.00	85.00	61.58	10.62
Fear of COVID 19	FCV19	7.00	38.00	26.09	7.99

The results of Pearson correlation showed that the turnover intention had a significant relationship with all the studied variables (P < 0.05). The highest positively correlation coefficient with the variable of turnover intention was related to job stress (0.408). The results also showed that all variables were significantly associated with job stress. Moreover, there was the highest negative correlation coefficients between job stress and general health status (− 0.431). In addition, based on the results, the studied variables had significant correlations with resilience. The highest negative correlation coefficient was found between resilience and job stress (-0.436) (see Table 3).

**Table 3:** Correlation matrix of the studied variables

Variable	General health	Mental workload	Work – family conflict	Fear of COVID 19	Job stress	Resilience	Turnover intention
General health	-						
Mental workload	0.012	-					
Work – family conflict	− 0.111	0.127^*^	-				
Fear of COVID 19	− 0.145^*^	0.216^**^	0.188^**^	-			
Job stress	− 0.431^**^	0.228^**^	0.364^**^	0.326^**^	-		
Resilience	0.160^**^	− 0.163^**^	− 0.254^**^	− 0.251^**^	− 0.436^**^	-	
Turnover intention	− 0.334^**^	0.122^*^	0.232^**^	0.191^**^	0.408^**^	− 0.256^**^	-

**Fig. 1:**
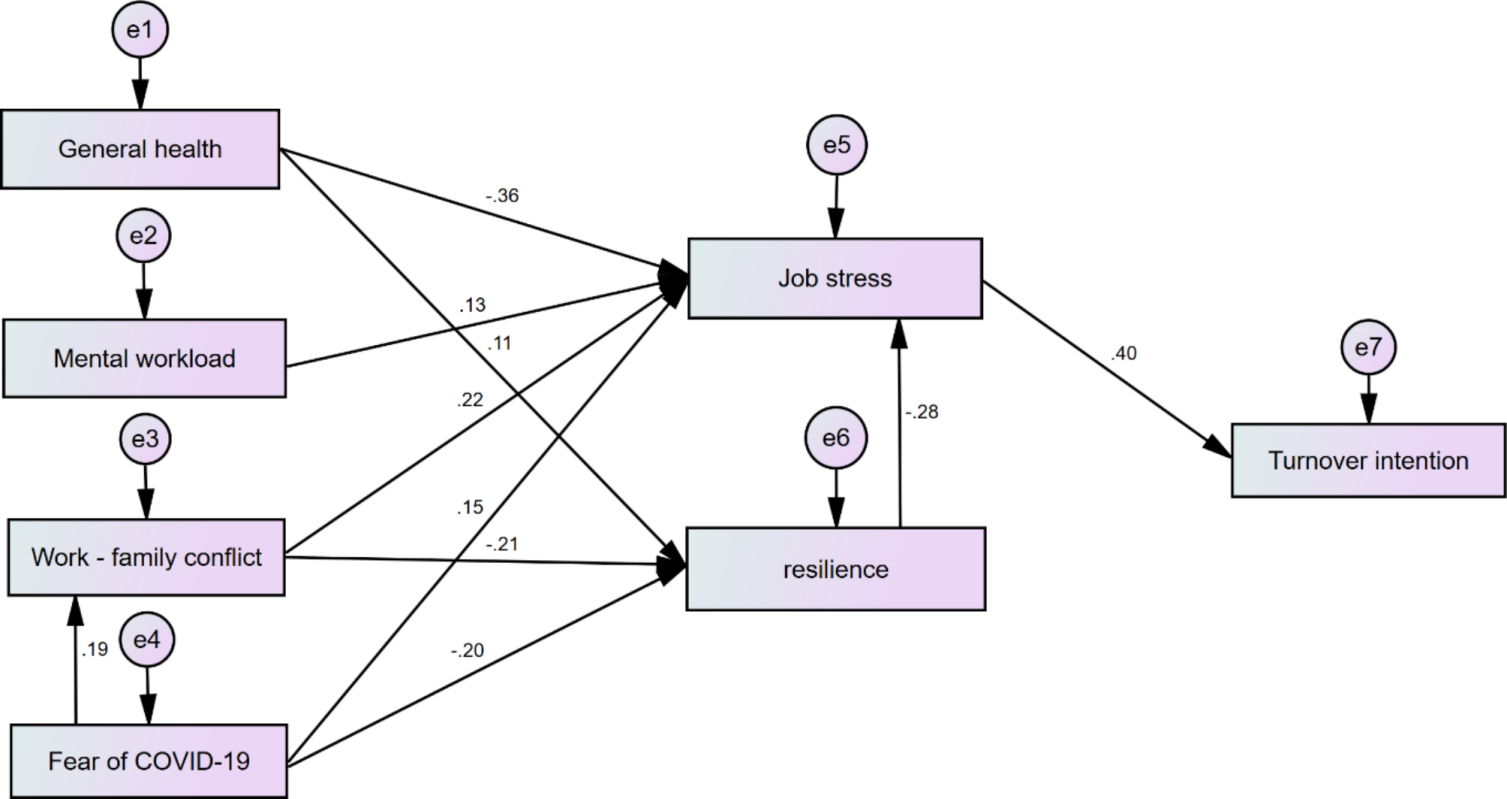
The drawn model for investigating the relationship between the variable

Figure 1 shows the model illustrating relationships between the studied variables. Table 4 also reports the standard path coefficients between the variables in the drawn model. The results showed that the four independent parameters of decreasing general health, increasing mental workload, increasing WFCs and fear of COVID-19 can indirectly increase nurses’ turnover intention by increasing job stress. Among these variables, the highest indirect effect coefficient on desire to leave the job was related to the general health parameter (-0.141). The findings also indicated an inverse relationship between people’s resilience and their level of job stress, with reduced resilience increasing job stress and, in turn, indirectly increasing turnover intention. Among the independent variables studied, three variables of decreasing general health, increasing work-family conflict and increasing fear of COVID-19 increased job stress level by reducing resilience and consequently increasing the turnover intention. The highest indirect effect factor in this way was related to the WFCs parameter (0.023). In addition, the results showed that fear of COVID-19 with coefficient of 0.188 directly affects WFCs. Table 5 also shows the fit indices of the drawn model. Based on these indicators, the model fits are approved.

**Table 4:** Standard path coefficients between variables in the model

Role of the variable Independent → dependent	Standard path coefficient	Standard error	P value
Fear of COVID 19 → Work–family conflict	0.188	0.075	< 0.001
General health → Job stress	− 0.357	0.147	< 0.001
Mental workload → Job stress	0.135	0.302	0.003
Work – family conflict → Job stress	0.222	0.177	< 0.001
Fear of COVID 19 → Job stress	0.145	0.235	0.002
General health → Resilience	0.109	0.101	0.045
Work–family conflict → Resilience	− 0.206	0.120	< 0.001
Fear of COVID 19 → Resilience	− 0.198	0.159	< 0.001
Resilience → Job stress	− 0.279	0.084	< 0.001
Job stress → Turnover intention	0.396	0.018	< 0.001

**Table 5:** The fit indices of the model

Indices	Name	Fitness	Obtained value
Absolute fitness indices	Goodness-of-fit index (GFI)	> 0.9	0.957
	Adjusted goodness-of-fit index (AGFI)	> 0.9	0.933
Comparative fitness indices	Normed fit index (NFI)	> 0.9	0.926
	Comparative fit index (CFI)	> 0.9	0.968
	Incremental fit index (IFI)	0–1	0.971
Normed fit index	Root mean squared error of approximation (RMSEA)	< 0.1	0.056
	Normed Chi-square (X^2^/df)	1–3	1.950

## Discussion

In this study, the effects of seven mental parameters such as general health, mental workload, work-family conflict and fear of COVID-19 on the turnover intention of 300 nurses was investigated with consideration of the role of job stress and resilience in the COVID-19 pandemic. According to the study’s findings, all of the variables were significantly correlated with turnover intention. General health and resilience had negative correlations with the turnover intention and mental workload, work – family conflict, fear of COVID-19, and job stress had positive correlations with the turnover intention. The results of path analysis showed that mental factors (including general health, mental workload, work – family conflict, and fear of COVID-19) through two paths can increase the intention to leave work. In the first path, decreasing general health, increasing mental workload, WFCs and fear of COVID-19 increase the turnover intention by increasing stress, and in the second path, mental factors (including general health, mental workload, work – family conflict, and fear of COVID-19) increase the turnover intention by reducing resilience.

The results of a study performed by Chieh et al. showed that the general health and job stress were associated with a reduction in work-related injury [44]. Yoon et al. also concluded that there is a significant relationship between mental health status and job stress contents among hospital nurses [45]. Qureshi et al. also studied the relationship between job stress, workload, and turnover intentions in the employees and concluded that employee turnover intentions are positively associated with job stressor and work load [46]. The results of a study performed by Junaidi et al. showed that that overtime, workload, and job stress can significantly influence the turnover intention [47]. Moreover, pandemic-related stressors appear to be increasing the mental workload of nurses as a result of psychological pressures, which in turn elevates stress levels and ultimately increases the turnover intention. Therefore, it is necessary to consider the workload and psychological well-being of nurses [48]. Furthermore, the results of a study conducted by Ahuja et al. revealed that balance of work-family conflict can impress on turnover intension [49]. The results of a study performed by Lu et al. indicated that there are the relationship between work–family conflict, work load, and work stress with turnover intention [50]. In addition, Labrague et al. conducted a study in 2021 to examine the effect of COVID-19 fear on job stress and turnover intention of Filipino nurses. The results showed that fear of COVID-19 has a significant positive correlation with stress and turnover intention [17]. Since nurses are highly likely to get COVID-19 due to direct exposure to Covid patients, the fear of getting infected or transmitting the virus to family members and friends leaves nurses overstressed, and ultimately can lead to nurses’ tendency to quit their professions or jobs [51, 52]. The results of a study performed by Santos et al. showed that nurses’ fears of COVID-19 increase their stress and can affect their turnover intention [53].

The findings also indicated an inverse relationship between people’s resilience and their level of job stress, with reduced resilience increasing job stress and, in turn, indirectly increasing turnover intention. The resilience in people can reduce the adverse consequences due to the unfavorable conditions and stress. Therefore, it implies that as resilience decreases, so does the amount of stress. Albert W. Wu and Kristen Santarone also concluded that resilience had the positive role in reducing stress and other mental disorders, such as anxiety and fear caused by COVID-19 [54, 55]. Also, the results of others study show that the more resilient workers are, the less likely they suffer symptoms of depression, anxiety, and stress [4].

Maintaining the physical and mental health of nurses during the COVID-19 pandemic period has high importance [56]. Nurses are one of the groups to bear the most occupational stress in difficult and critical conditions and play the main role in the management of infectious diseases, particularly in the pandemic conditions. When the level of job pressures on nurses increases, they try to deal with stressful situations in different ways [57]. The weakened job performance (voluntary or involuntary) is one of ways for decreasing this stress. It can be due to this fact that the person in stressful conditions consume a part of their strength and energy to deal with stressful factors [58]. In these conditions, people have the limitation of power and energy to perform the desired task. If these conditions continue, the level of person’s performance will be weakened, and it may be associated with other negative consequences. For this reason, people may try to retain their performance under the stressful conditions [59]. From this point of view, consequences such as the tendency to turnover is expectable.

The retention of human resources as valuable capital of the organization has gained importance among managers and employers over recent years. The findings of the present study can be used as a guide for decision-making and human resource management in order to reduce the tendency of nurses to leave their jobs in hospitals. Working in difficult and exhausting conditions such as the epidemic of COVID-19, which is accompanied by a lot of stress, can increase the desire to leave the service in nurses. The most important solution in order to reduce the desire to leave the service in nurses is to increase the resilience of people in critical situations and implement resilience training programs. To buffer these issues and boost nurses’ mental and physical resilience, it is necessary to plan and implement coping management programs. According to WHO guidelines, provision of coping skills including adequate sleep, eating a healthy and balanced diet, regular exercise, staying in touch with friends and family, and receiving administrative, organizational, and social support can play a key role in this regard [60]. Murat et al. investigated the effect of COVID-19 coping strategies on the relationship between COVID-19 anxiety and general health. Findings showed that anxiety caused by COVID-19 has a negative effect on general health and coping with COVID-19 has significant direct effects on general health [4]. In previous studies, coping skill has been introduced as a remarkable contributing factor to the mental and physical health of individuals [61].

In total, although many studies have been conducted so far regarding the proof of mental complications caused by the COVID-19, there are still few studies conducting such analysis exploring that Covid pandemic can cause such complications. Different parameters can play a role in creating a mental disorder. By analyzing and understanding different paths, preventive solutions can be defined and implemented to reduce the severity and probability of these complications. The present study tried to evaluate and analyze the different paths that can lead to turnover intention in nurses during the COVID-19 pandemic. For future studies, it is suggested that factors and risk factors effective in causing or accelerating the occurrence of psychological complications among nurses should be identified and more advanced models should be developed using path analysis modeling or the use of Bayesian networks in order to analyze and determine more probable paths. To identify and priority the risk factor affecting mental complications, person can use decision-making methods, such as the fuzzy Delphi technique, hierarchical analysis method and process network analysis.

One of the limitations of the present study was that, although many variables may modify the effect of mental factors on turnover intensions, only two variables of job stress and resilience were considered as modifiers. In this study, seven scales only entered into the model. Furthermore, given that the questionnaires were electronically completed by participants, there may be a response bias compared to face-to-face interview. Moreover, demographic variables also may influence the turnover intention. However, given the limitation of sample number, only the main variables were entered into the model for obtaining the proper fitness of model. Therefore, it was suggested that the effect of these variables is studied in the next researches.

## Conclusion

Overall, the results showed that the four independent parameters of decreasing general health, increasing mental workload, increasing WFCs and fear of COVID-19 can indirectly increase nurses’ turnover intention by increasing job stress. Of these factors, the greatest effect on turnover intention was related to the general health. Based on the results, there are an inverse relationship between people’s resilience and their level of job stress. Therefore, it is expected that if frontline nurses are trained and equipped with adequate levels of psychological resilience, they are more likely to survive challenges at work and sustain their clinical performance and hence foster their job retention.

## Data Availability

The datasets used and/or analyzed during the current study are available from the corresponding author on reasonable request.
